# Is It Possible to Shorten the Duration of Adjuvant Chemotherapy for Locally Advanced Rectal Cancer?

**DOI:** 10.1097/MD.0000000000003427

**Published:** 2016-04-22

**Authors:** Kai-Yun You, Rong Huang, Xin Yu, Yi-Min Liu, Yuan-Hong Gao

**Affiliations:** From the Department of Radiation Oncology (K-YY, Y-ML), SunYat-Sen Memorial Hospital, SunYat-Sen University; and Department of Radiation Oncology (RH, XY, Y-HG), State Key Laboratory of Oncology in South China, Sun Yat-sen University Cancer Center, Collaborative Innovation Center of Cancer Medicine, Guangzhou, China.

## Abstract

The long duration of 4 months of postoperative adjuvant chemotherapy is currently recommended for locally advanced rectal cancer after preoperative chemoradiation and surgery. Whether a short duration could be applied in these patients is unknown. So, the purpose of this study is to evaluate the effects on prognosis based on different durations of adjuvant chemotherapy for rectal cancer.

We performed a retrospective study of 200 rectal cancer patients who were treated with preoperative chemoradiation and were pathologically graded as ypII and ypIII stages between March 2003 and May 2012. All patients were divided into 2 groups according to the median duration of adjuvant chemotherapy of 2 months. Overall survival (OS) and disease-free survival (DFS) were compared between patients with duration shorter and longer than 2 months in the whole group and subgroups of ypII and ypIII. Recurrence patterns were also analyzed in all subgroups. Multivariate analysis was performed to explore clinical factors that were significantly associated with DFS, local recurrence-free survival, and distant metastasis-free survival.

In subgroup of ypII stage, the 5-year OS and DFS were similar between patients in long and short durations of adjuvant chemotherapy. For patients of ypIII stage, although no significant difference was found in OS between patients in short and long durations, DFS was showed to be higher in the group of long duration. Further analysis showed that longer duration of adjuvant chemotherapy could lead to improved control of distant metastasis and no impact on local control. Multivariable analysis indicated that long duration of adjuvant chemotherapy is significantly associated with longer distant metastasis-free survival in patients with ypIII stage, but not in those with ypII stage.

A long duration of at least 2 months of postoperative adjuvant chemotherapy is necessary for patients with ypIII stage, whereas it may not be absolutely appropriate for those with ypII stage. Therefore, we suggest a tailored selection of durations of adjuvant chemotherapy for locally advanced rectal cancer.

## INTRODUCTION

Currently, the standard care for locally advanced rectal cancer is preoperative chemoradiotherapy followed by surgery. Irrespective of the final pathology, approximately 4 months of postoperative adjuvant chemotherapy (total 6 months of perioperative chemotherapy) is typically recommended.^1^ However, the evidence supporting this recommendation is limited.^2^ Several studies have questioned the need of adjuvant chemotherapy for locally advanced rectal patients, especially for those with ypT0–2,^3–6^ a subset of patients who exhibited favorable response and were reported to achieve excellent survival regardless of receiving adjuvant chemotherapy or not.^7–9^

Meanwhile, the recent clinical trial of ADORE has well established the significant value of FOLFOX adjuvant chemotherapy for rectal cancer with ypII-III.^10^ However, the percentage of patients who could fulfill the full courses of adjuvant chemotherapy is less than 50% and many of the patients are unable to complete all the planned courses due to the side effects or other reasons during the long time of adjuvant chemotherapy.^3,11,12^ Thus, some patients received just inadequate durations of postoperative adjuvant chemotherapy. But, whether a shorter duration of adjuvant chemotherapy is suitable for rectal cancer patients is still unknown. Previous work has proved that adjuvant chemotherapy may not be need for ypT0–2N0 patients, whereas there were still some controversies regarding patients with ypT3–4N0.^4,10^ And our present study is to further explore whether a shorter duration of adjuvant chemotherapy should be adopted for the patients with ypT3–4N0, and also for those with ypTanyN+.

## METHODS

### Ethics Statement

This research was approved by the Ethics Committee of Sun Yat-sen University Cancer Center, and written informed consent was obtained from every patients included in the study.

### Patients

The data were extracted from a prospective database that enrolled all patients who underwent surgical treatment at Sun Yat-sen University Cancer Center from 2004 to 2012. The patient characteristics, operative findings, pathologic reports, adjuvant treatment, and follow-up data were included in the database. The selection criteria for the study were as follows: locally advanced rectal cancer, staged based on a clinical examination, such as endorectal ultrasound, pelvic computed tomography, and magnetic resonance imaging; received preoperative chemoradiation followed by total mesorectal excision (TME) surgery; pathologically staged as ypII and ypIII; underwent at least 1 course of postoperative adjuvant chemotherapy; no evidence of distant metastasis during the treatment and no concurrent malignancy or prior history of radiotherapy to the pelvis. Two hundred cases were included after reviewing the clinical data.

### Treatment

All the patients were prescribed to receive a standard protocol of neoadjuvant chemoradiotherapy, including 2 courses of concurrent chemotherapy. The prescribed dose for the entire pelvis was 46 Gy in 23 fractions for the pelvic with an additional 4 Gy divided into 2 fractions injected into the primary tumor. The radiotherapy technique was based on a 3-dimensional conformal radiotherapy treatment planning system (PINNACLE 8) using a 3-field irradiation plan (8-MV photon posterior–anterior field and 15-MV photon opposed lateral beams). The clinical target volume (CTV) has included the region of primary rectal tumor, perirectal tissues, the presacral lymph nodes, the internal iliac lymph nodes, and the obturator lymph nodes. The superior border of the CTV was at the bottom of L5, and the inferior border was 3 cm distal to the tumor. The anterior border was the posterior margin of the bladder or uterus, and the posterior border was the anterior margin of the sacrum. Planned target volume was defined as CTV+ 8 to 10 mm.

The regimens of the concurrent chemotherapy were FOLFOX6 and Xelox. A total of 41 patients were treated with chemotherapy using FOLFOX6 (85 mg/m^2^ oxaliplatin, 400 mg/m^2^ leucovorin, and 400 mg/m^2^ 5-FU iv d1 followed by 2400 mg/m^2^ civ 46–48 hours), and the other 159 patients received Xelox (100 mg/m^2^ oxaliplatin d1 and 1000 mg/m^2^ capecitabine bid, po, d1–14).

At an interval of 5 to 12 weeks after the completion of chemoradiotherapy, radical surgery for rectal cancer was conducted. All operations were performed by colorectal surgeons according to the principles and methods of TME, including low anterior resection, abdominoperineal resection, and Hartmann.

Adjuvant chemotherapy was administered to the patients according to the regimens of FOLFOX6 (85 mg/m^2^ oxaliplatin, 400 mg/m^2^ leucovorin, and 400 mg/m^2^ 5-FU iv d1 followed by 2400 mg/m^2^ civ 46–48 hours), Xelox (130 mg/m^2^ oxaliplatin d1 and 1000 mg/m^2^ capecitabine bid, po, d1–14), or the single agent capecitabine (1250 mg/m^2^ bid, po, d1–14). The median duration of adjuvant chemotherapy was 2 months (range 0.7–4.2 months). Thus, all the patients included in our study were dichotomized into 2 groups according to the median duration: short-duration group and long-duration group.

### Pathologic Classification

Pathologic tumor staging of the resected specimen was performed by experienced pathologists. The operative specimens of 200 patients were restaged according to the American Joint Committee on Cancer (AJCC) 7th Edition staging system. All of the specimens were carefully dissected to evaluate all potentially involved lymph nodes, and the median number of retrieved lymph nodes was 6 (range 2–37 nodes). Additionally, circumferential resection margin (CRM) involvement was defined as the maximum distance between the tumor and the proper rectal fascia of less than 1 mm. Negative CRM, as demonstrated by pathology, was achieved for all patients in this study.

### Toxicity Assessment for Adjuvant Chemotherapy

The therapy-related adverse events were defined as complications that occurred during adjuvant treatment, which were graded based on Cancer Institute Common Terminology Criteria for Adverse Events, version 3.0. Severe adverse events were defined as any grade ≥3 toxicity. Adverse events were recorded for each patient and were documented in our colorectal database**.**

### Follow-up

The follow-up policy was every 3 months for the first 2 years after surgery and every 6 months thereafter. The evaluations were done based on the complete blood count, liver function test, carcinoembryonic antigen (CEA), carbohydrate antigen 19–9 (CA19–9), and physical examination during each visit. Chest radiography, computed tomography scanning of the abdomen and pelvis, and colonoscopy were conducted every 6 months. The follow-ups for each patient were recorded in our database. In this study, the median follow-up period for all patients was 46 (range 10–107) months.

### Statistical Analysis

All statistical analyses were performed by SPSS software, version 18.0. Categorical variables were analyzed by using the chi-square test or Fisher exact test. Continuous variables were analyzed by the Student *t* test or Mann–Whitney *U* test. The Kaplan–Meier method was employed to compare disease-free survival (DFS) rates and overall survival (OS) rates. Multivariate analysis of DFS, local recurrence-free survival (LRFS), and distant metastasis-free survival (DMFS) was performed by Cox proportional-hazards regression, and Cox proportional-hazards model was performed using a forward conditional selection of variables. Variables with *P* value <0.2 were entered into a Cox model. *P *< 0.05 was considered to be statistically significant.

## RESULTS

### Clinical Characteristics

In all, 200 patients who underwent chemo-radiotherapy treatment and radical resection were included in our study. Among these patients, 120 patients were administered with 2 to 4.2 months of adjuvant chemotherapy, and the remaining 80 patients underwent it for a shorter duration of 0.7 to 2 months. Comparing with the patients who have received a longer duration of adjuvant chemotherapy, those who have received a shorter duration of it exhibited an older appearance, lower level of pretreatment CEA, and more percentage of clinical II stage. No significant difference in sex, hemoglobin (Hb), tumor location, clinical T stage, clinical N stage, tumor grade, concurrent chemotherapy, type of surgery, number of retrieved lymph nodes, ypN stage, ypT stage, regimens of adjuvant chemotherapy, and follow-ups were showed between the 2 groups (Table 1).

**TABLE 1:**
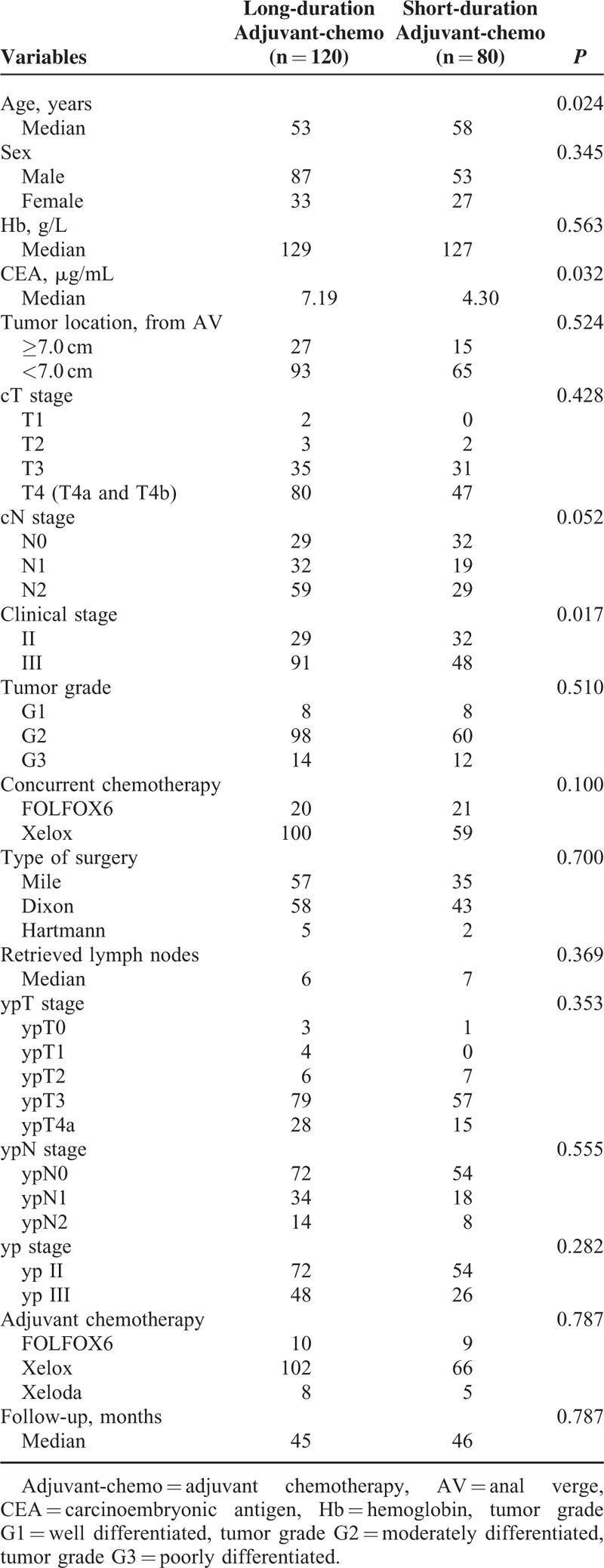
Patient Demographics, Baseline Tumor Characteristics, Type of Surgery, and Pathologic Outcome

### Survival Analysis for the Entire Group

**FIGURE 1:**
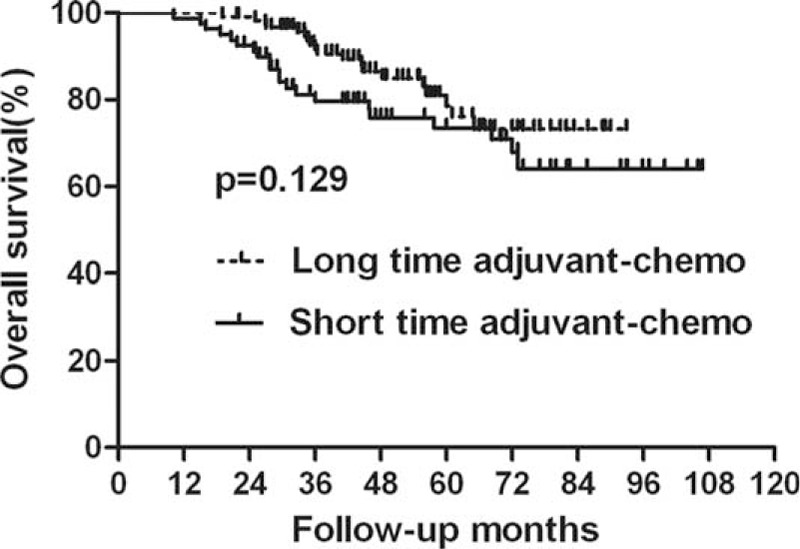
Overall survival (OS) for the whole group stratified by duration of adjuvant chemotherapy. No significant difference was found in OS between patients with long and short durations for the whole group (*P* = 0.129).

**FIGURE 2:**
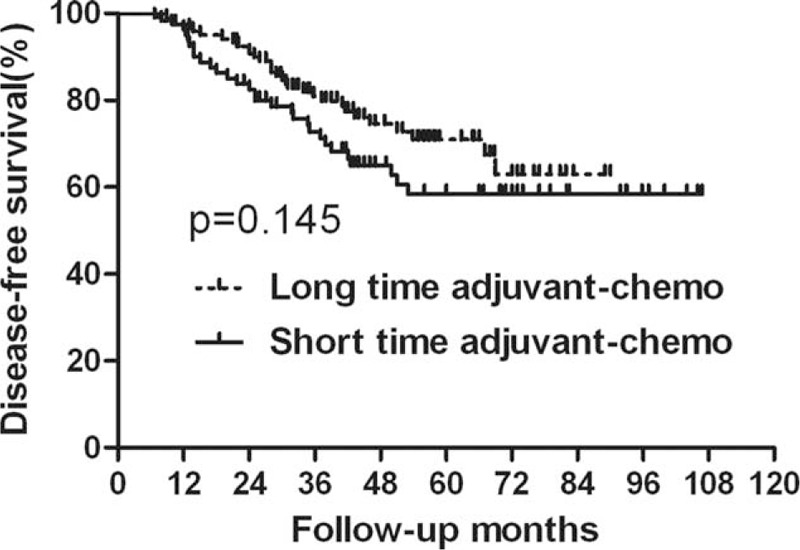
Disease-free survival (DFS) for the whole group stratified by duration of adjuvant chemotherapy. No significant difference was found in DFS between patients with long and short durations for the whole group (*P* = 0.145).

For the entire group, 40 patients died during the follow-ups. The 5-year OS rates in the long-duration and short-duration groups were 78.5% and 73.5%, respectively (Figure 1, Table 2), which was not significantly different between the 2 groups (*P* = 0.129). Sixty patients with tumor recurred. Among them, local recurrence was found in 18 patients, distant metastasis was detected in 32 patients, and both local and distant recurrences were found in 10 patients. Furthermore, the 5-year DFS rates were also comparable between the long-duration and short-duration groups (71.0% vs 58.5%; *P* = 0.145) (Figure 2, Table 2). We also performed the analysis based on ypTNM stage, and it was found that patients with ypIII stage acquired worse survival than those with ypII stage (Table 3).

**TABLE 2:**
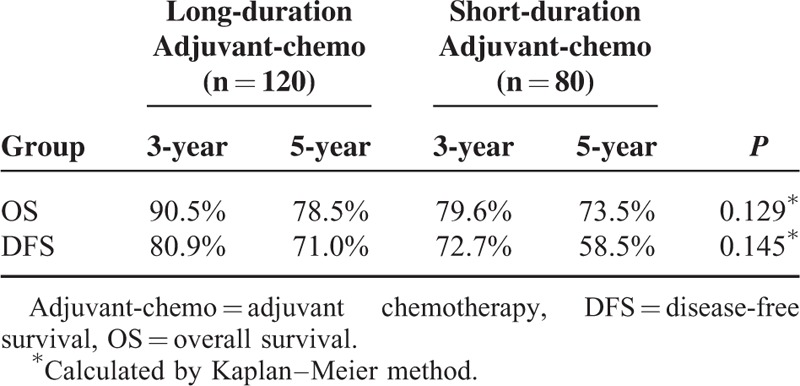
Survival for the Whole Group Patients

**TABLE 3:**
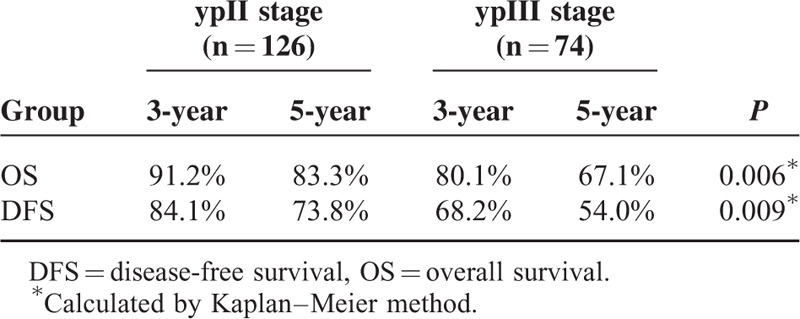
The Comparison of the OS and DFS between ypII Stage and ypIII Stage

### Survival Analysis for the Subgroup of ypII Stage

**FIGURE 3:**
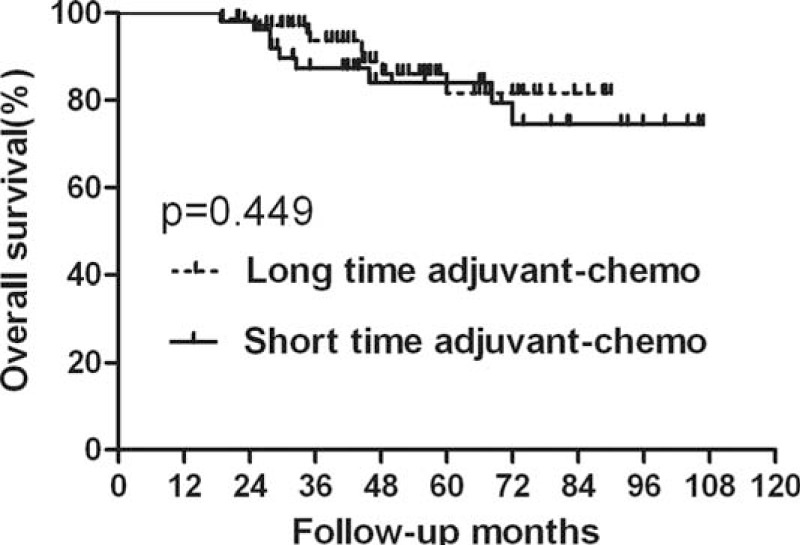
Overall survival (OS) for the subgroup of ypII stage stratified by duration of adjuvant chemotherapy. No significant difference was found in OS between patients with long and short durations for subgroup of ypII stage (*P* = 0.449).

**FIGURE 4:**
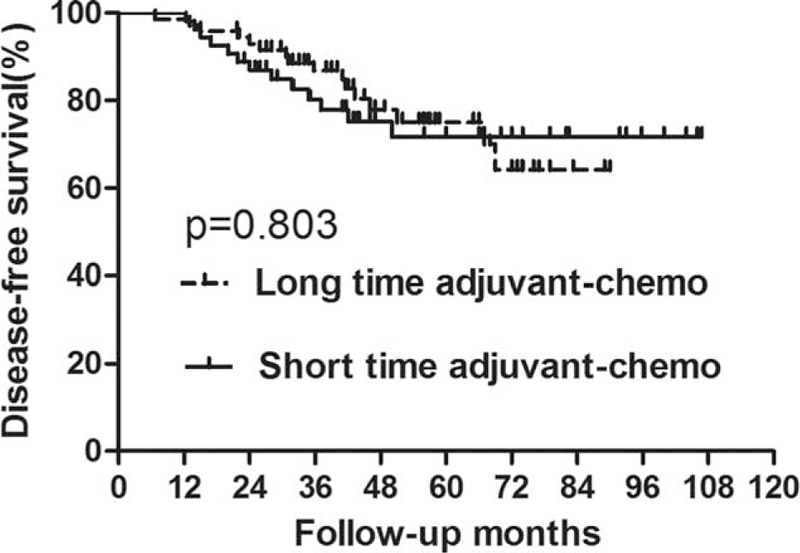
Disease-free survival (DFS) for the subgroup of ypII stage stratified by duration of adjuvant chemotherapy. No significant difference was found in DFS between patients with long and short durations for subgroup of ypII stage (*P* = 0.803).

In the subgroup of ypII stage, 29 patients experienced recurrence; among these 17 patients died of tumor recurrence. There were 9 patients who displayed local recurrence and 16 patients who only exhibited distant metastasis. The remaining 4 patients developed both local and distant recurrence. In the long-duration and short-duration groups, the 5-year OS rates were 81.6% and 84.1%, respectively, and the 5-year DFS rates were 75.0% and 71.8%, respectively (Figures 3 and 4, Table 4). No significant difference was detected in either OS (*P* = 0.409) or DFS (*P* = 0.803). Further analysis of the recurrence pattern revealed that there were no differences in both the local recurrence and distant metastasis rates between groups of longer duration and shorter duration (*P* > 0.05) (Table 5).

**TABLE 4:**
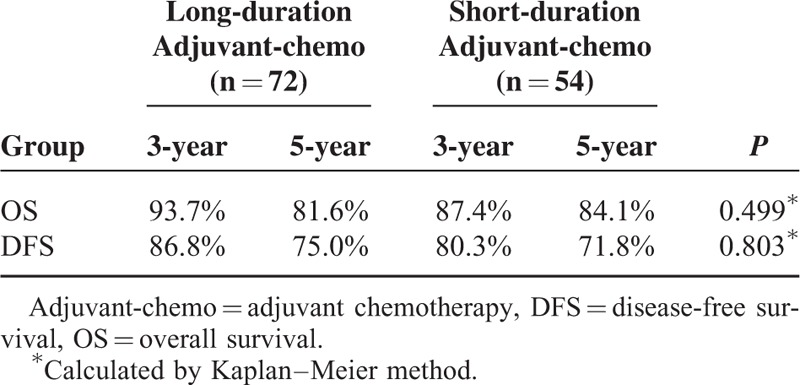
Survival for Subgroup of ypII Stage

**TABLE 5:**
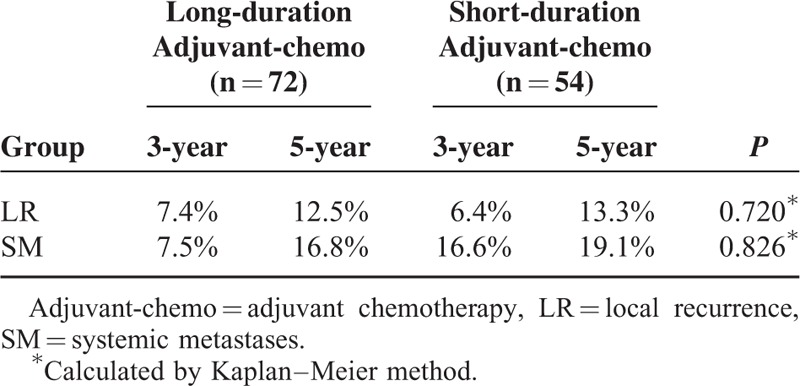
Recurrence Patterns for Patients with ypII Stage

### Survival Analysis for the Subgroup of ypIII Stage

**FIGURE 5:**
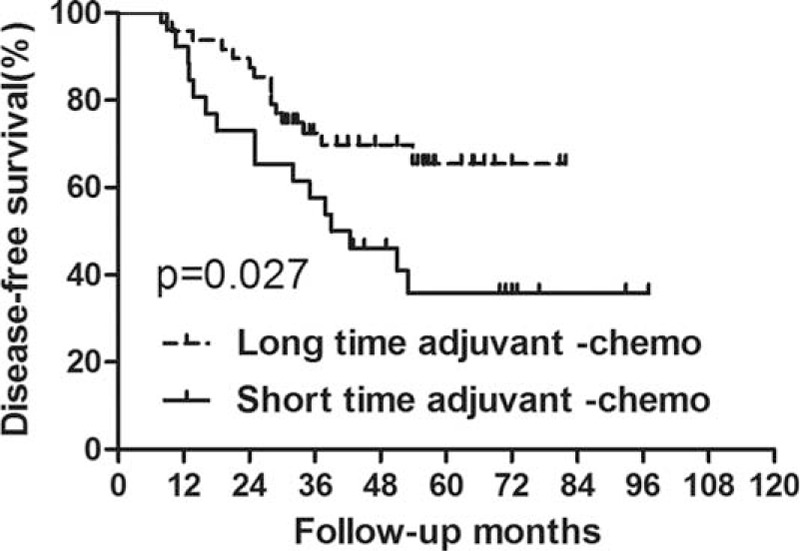
Disease-free survival (DFS) for the subgroup of ypIII stage stratified by duration of adjuvant chemotherapy. Significant difference was found in DFS between patients with long and short durations for subgroup of ypIII stage (*P* = 0.027).

**FIGURE 6:**
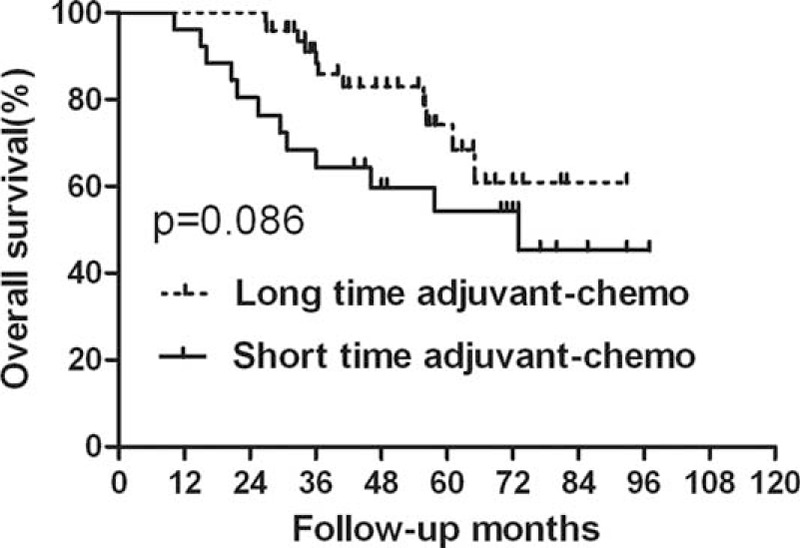
Overall survival (OS) for the subgroup of ypIII stage stratified by duration of adjuvant chemotherapy. No significant difference was found in OS between patients with long and short durations for subgroup of ypIII stage (*P* = 0.086).

For the patients with ypIII stage, those who were undergoing long duration of adjuvant chemotherapy exhibited longer DFS than those who were undergoing short duration of it (*P* = 0.027) (Figure 5, Table 6). However, there was no significant difference in OS between the 2 groups (*P* = 0.086) (Figure 6, Table 6). Additionally, based on analysis of distant metastasis, the patients in the long-duration adjuvant-chemo group displayed a lower rate of distant metastasis than those in the short-duration adjuvant-chemo group (*P* = 0.041). Meanwhile, the rate of local recurrence between the 2 groups was similar (*P* = 0.364) (Table 7).

**TABLE 6:**
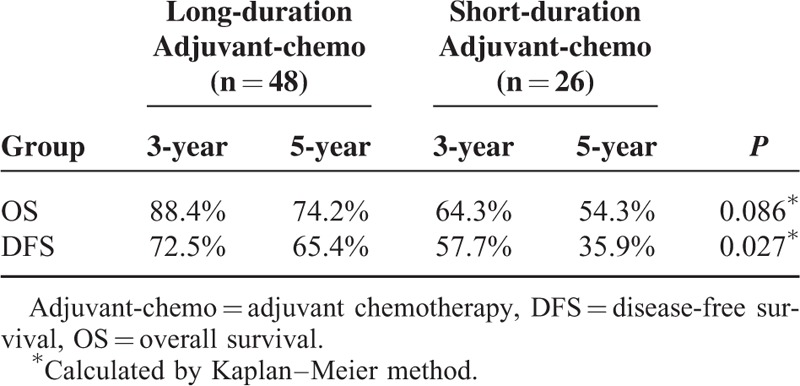
Survival for Subgroup of ypIII Stage

**TABLE 7:**
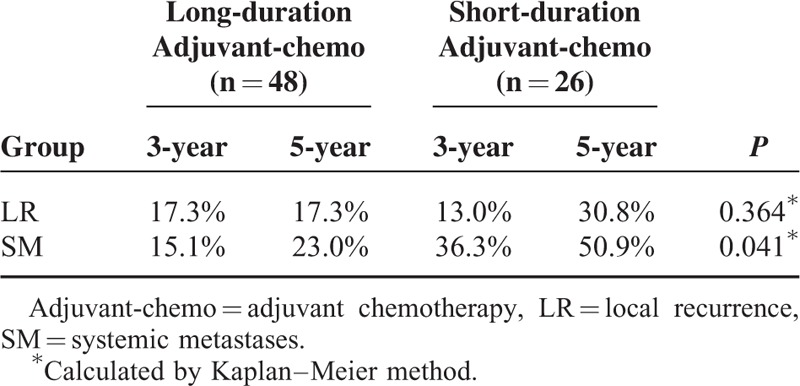
Recurrence Patterns for Patients with ypIII Stage

### Clinical Predictors of DFS, LRFS, and DMFS

For patients with ypIII stage, multivariate analysis indicates that the factors of adjuvant chemotherapy and retrieved lymph nodes were the independent predictors of DFS. And the retrieved lymph nodes were found to be specifically associated with LRFS. Furthermore, short-duration adjuvant chemotherapy was also found to be significantly associated with lower DMFS, with patients who have received short-duration adjuvant chemotherapy having a 2.4-fold increased risk of distant metastasis in relation to those who received long duration of it (Table 8). However, in patients with ypII stage, the duration of adjuvant chemotherapy was not significantly associated with the survival. The location of the tumor was found to be the only factor to predict DMFS, and no other factors were detected to be associated with DFS or LRFS (Table 9).

**TABLE 8:**
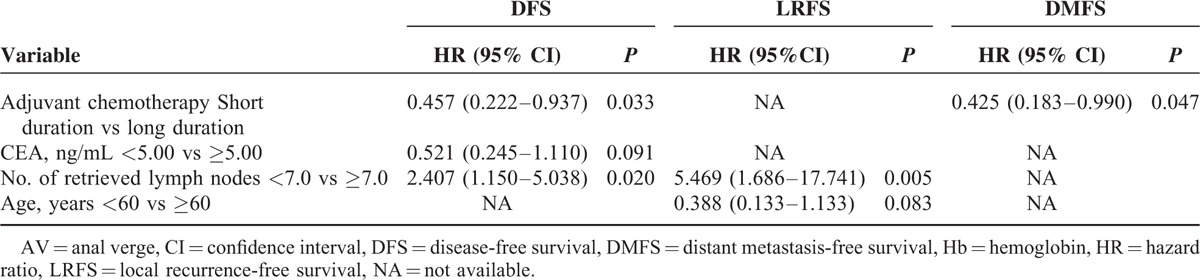
Multivariate Analyses of DFS, LRFS, and DMFS for ypIII Stage Patients

**TABLE 9:**
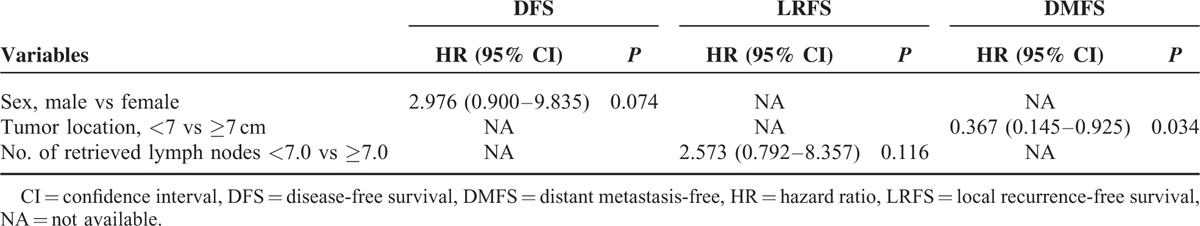
Multivariate Analyses of DFS, LRFS, and DMFS for ypII Stage Patients

### Toxicity of Adjuvant Chemotherapy

The most common toxicity types for adjuvant chemotherapy were diarrhea, hand-foot syndrome, and neutropenia. Severe adverse events during the adjuvant chemotherapy were observed mostly in the toxicity types of diarrhea, hand-foot syndrome, and nausea. It was further found that severe adverse events of toxicity types of diarrhea and hand-foot syndrome occurred more likely in the group receiving long-duration therapy than in the group receiving short-duration therapy. Whereas, other toxicity types with severe grade (grade ≥3) were similar between the 2 groups (Table 10).

**TABLE 10:**
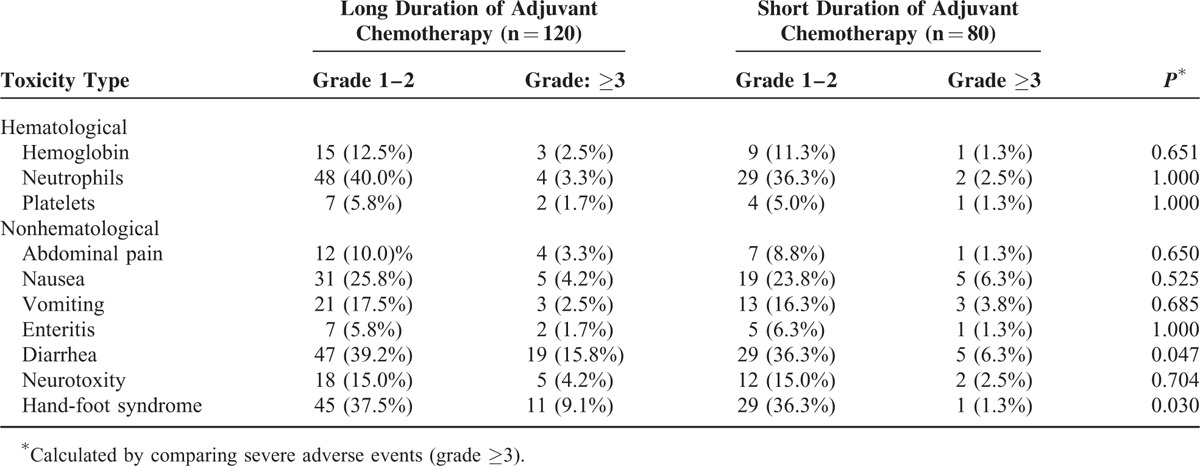
Toxicity of Adjuvant Chemotherapy

## DISCUSSION

Although a full course of adjuvant chemotherapy is still highly recommended for locally advanced rectal cancer after long-course chemoradiotherapy followed by radical resection, our current study has proposed certain challenges to this process of care. We found out that there were no significant differences in both OS and DFS among the patients who received long and short duration of adjuvant chemotherapy for the whole group of patients in our study. However, further subgroup analysis showed that, in patients with ypIII stage, those who were in the long-duration adjuvant-chemo group acquired longer DFS than those who were in the short-duration adjuvant-chemo group. But, in the subgroup of ypII stage, better survival has not been detected in patients who have received longer duration of adjuvant chemotherapy compared with those who have received shorter duration of it. Furthermore, we explored the pattern of recurrence for different subgroups. In the subgroup of ypIII stage, the results indicated similar local recurrence rates between patients who received long and short duration of adjuvant chemotherapy, but the rate of distant metastasis was found to be higher in the short-duration adjuvant-chemo group than that in the long-duration adjuvant-chemo group. Most importantly, this decreased rate of distant metastasis brought by longer duration of adjuvant chemotherapy was not shown in patients with ypII stage, revealing that the benefit of the long-duration postoperative adjuvant chemotherapy was focused on reducing the risk of distant metastasis only for those with ypIII stage. The value for patients with ypII stage was not fully supported. Multivariate analysis was performed using Cox proportional-hazards regression to ensure that the results are presented in a more constructed manner, The results showed that adjuvant chemotherapy was an independent predictor of DMFS for patients with ypIII stage, with patients who have received shorter duration of adjuvant chemotherapy having a 2.4-fold increased risk of distant metastasis compared with those who have received a longer duration of it. However, for patients with ypII stage, adjuvant chemotherapy was not significantly associated with the survival.

The benefit of adjuvant chemotherapy after neoadjuvant chemoradiotherapy and surgery in locally advanced rectal cancer is controversial and therefore, may be a reason for the wide variability in applications of adjuvant chemotherapy among institutions.^2,13^ There is also no conclusive evidence to define the optimal duration and regimen of adjuvant chemotherapy, which may lead to variability in physician recommendations and the patients’ decisions from different trials.^10–12,14–16^ As we know, 4 randomized clinical trials have specifically evaluated the value of adjuvant chemotherapy for locally advanced rectal cancer receiving neoadjuvant chemoradiotherpy. And they are EORTC22921, CHRONICLE, I-CNR-RT, and PROCTOR-SCRIPT trials.^11,14–16^ None of the 4 trials showed significant differences of OS and DFS between the patients who did and did not receive postoperative adjuvant chemotherapy. However, before we may accept the conclusion, we should also notice that the compliance to adjuvant chemotherapy was poor in those trials. Less than 50% of the patients in the EORTC22921 trial and CHRONICLE trial completed all cycles of chemotherapy. Thus, we could not exclude the possibility that it was due to the inadequate duration of adjuvant chemotherapy in the experimental group that lead to no improvement in survival by the adjuvant treatment. This hypothesis may be supported by the trial of ADORE, in which nearly all the patients completed the long-duration adjuvant chemotherapy and FOLFOX adjuvant chemotherapy was found to be effective. In the trial of ADORE, Hong et al randomly assigned 321 patients to use fluorouracil + leucovorin and FOLFOX. After a median follow-up of 38.2 months, the 3-year DFS was shown to be significantly higher in the FOLFOX group compared with that in fluorouracil + leucovorin group. And this difference was proved to be more obvious in patients with pathological stage III.^10^ The ADORE trial just indicated that adjuvant chemotherapy of FOLFOX was effective in rectal cancer patients with ypII and ypIII, and the regimen of adjuvant chemotherapy added with oxaliplatin tend to bring better survival than that containing only fluorouracil and leucovorin. Besides, Hong and Ryan mentioned that the reduced-dose bolus fashion of postoperative chemotherapy in the EORTC22921 trial, the small numbers of patients and poor accrual in CHRONICLE trial, and the underpower to detect a small survival benefit in the PROCTOR-SCRIPT trial may also affect the true results of the trials which were designed to explore the need of adjuvant chemotherapy for locally advanced rectal cancer.^17^ Furthermore, some studies still supported the routine use of postoperative adjuvant chemotherpay in some subsets of rectal cancer patients, especially in patients with ypII and ypIII stage.^18,19^ However, whether a short-duration adjuvant therapy could be applied is still unknown. Our present work further addresses this problem, showing that a short duration of fewer than 2 months of adjuvant therapy did not lead to impaired survival for patients with ypII stage, and long-duration adjuvant chemotherapy may still be needed for patients with ypIII stage.

The clinical dilemma of low compliance to the long duration of 4 months of postoperative adjuvant chemotherapy is often faced by the patients and physicians. Many patients failed to complete the long-duration adjuvant chemotherapy and some patients did not even receive it at all. As is reported by Haynes et al, significant variation exists in the receipt of postoperative chemotherapy after resection and it was shown that pathologic stage was the strongest determinant of which patients received postoperative chemotherapy with stage I are less likely to receive it than stage III.^20^ Other factors, such as age, Eastern Cooperative Oncology Group performance status ≥1, on Medicaid or indigent compared with private insurance, presence of reoperation/wound infection, and no closure of ileostomy/colostomy, were also found to be significantly associated with not receiving adjuvant chemotherapy.^21^ The reasons accounted for not completing all courses of the adjuvant chemotherapy in our study were mainly the toxicity caused by chemotherapy, poor performance status, the economic ability, and refusal. Whether shortening the adjuvant treatment time would be possible for these patients was clinically meaningful. As far as we know, we are the first to hypothesize and suggest that a short duration of adjuvant chemotherapy may be applied in patients with ypII stage. Whereas, for patients with ypIII stage, long duration of adjuvant chemotherapy seems to play a vital role in decreasing the rate of distant metastasis and should still be administered among these patients. Although our study was retrospective, there was no randomized trial that would specifically address this problem. The value was that many more patients with ypII stage would only undergo shorter duration of postoperative adjuvant chemotherapy, which can save a lot of time and alleviate the severity of toxicity caused by the chemotherapy.

There were several limitations in our present study. One of which was a retrospective analysis of a small sample size of rectal cancer patients with a short follow-up. Although we have supported that, in patients with ypII stage, long time of more than 2 months of adjuvant chemotherapy did not lead to better survival than that of fewer than 2 months, the exact duration has not yet been clearly outlined and it would only be fully answered by large randomized controlled clinical trials. Furthermore, patients with ypIII stage were more likely to develop distant failure rather than local recurrence in the long-term follow-up. Thus, whether the intensity of 4 months of postoperative adjuvant chemotherapy recommended by National Comprehensive Cancer Network guidelines could actually eradicate the potential of micro-distant metastasis is still unknown. Another major problem was due to the limited cases for patients receiving different regimens in the adjuvant chemotherapy, it prevented us from doing further analyses in deciding whether regimen added with oxaplatin would bring improved survival, as was found in the trial of ADORE.

In conclusion, the current study performed by us does not suggest more than 2 months of adjuvant chemotherapy for rectal cancer patients with ypII stage. However, for patients with ypIII stage, adjuvant chemotherapy of fewer than 2 months showed limited power to control the rate of distant metastasis. Thus, at least 2 months of adjuvant chemotherapy is highly recommended for these patients. Furthermore, distant failure was a major problem in locally advanced rectal cancer after preoperative chemoradiotherapy, and systemic therapy seems to be the main treatment to control it. So, identifying the optimum duration and regimen of adjuvant chemotherapy is imperative and need further investigation.

## References

[1] National Comprehensive Cancer Network. NCCN clinical practice guidelines in oncology: rectal cancer, 2015 http://www.nccn.org/. 10.6004/jnccn.2009.0057 19755047

[2] Bujko K Glynne-Jones R Bujko M Does adjuvant fluoropyrimidine-based chemotherapy provide a benefit for patients with resected rectal cancer who have already received neoadjuvant radiochemotherapy? A systematic review of randomised trials. *Ann Oncol* 2010; 21:1743–1750. 20231300 10.1093/annonc/mdq054

[3] Bosset JF Collette L Calais G Chemotherapy with preoperative radiotherapy in rectal cancer. *N Engl J Med* 2006; 355:1114–1123. 16971718 10.1056/NEJMoa060829

[4] Govindarajan A Reidy D Weiser MR Recurrence rates and prognostic factors in ypN0 rectal cancer after neoadjuvant chemoradiation and total mesorectal excision. *Ann Surg Oncol* 2011; 18:3666–3672. 21590450 10.1245/s10434-011-1788-y

[5] Huh JW Kim HR Postoperative chemotherapy after neo-adjuvant chemoradiation and surgery for rectal cancer: Is it essential for patients with ypT0–2N0? *J Surg Oncol* 2009; 100:387–391. 19582821 10.1002/jso.21342

[6] Kiran RP Kirat HT Burgess AN Is adjuvant chemotherapy really needed after curative surgery for rectal cancer patients who are node-negative after neoadjuvant chemoradiotherapy? *Ann Surg Oncol* 2012; 19:1206–1212. 21935748 10.1245/s10434-011-2044-1

[7] Capirci C Valentini V Cionini L Prognostic value of pathologic complete response after neoadjuvant therapy in locally advanced rectal cancer: long-term analysis of 566 ypCR patients. *Int J Radiat Oncol Biol Phys* 2008; 72:99–107. 18407433 10.1016/j.ijrobp.2007.12.019

[8] de Campos-Lobato LF Stocchi L Da LMA Pathologic complete response after neoadjuvant treatment for rectal cancer decreases distant recurrence and could eradicate local recurrence. *Ann Surg Oncol* 2011; 18:1590–1598. 21207164 10.1245/s10434-010-1506-1

[9] Park IJ You YN Agarwal A Neoadjuvant treatment response as an early response indicator for patients with rectal cancer. *J Clin Oncol* 2012; 30:1770–1776. 22493423 10.1200/JCO.2011.39.7901 PMC3383178

[10] Hong YS Nam BH Kim KP Oxaliplatin, fluorouracil, and leucovorin versus fluorouracil and leucovorin as adjuvant chemotherapy for locally advanced rectal cancer after preoperative chemoradiotherapy (ADORE): an open-label, multicentre, phase 2, randomised controlled trial. *Lancet Oncol* 2014; 15:1245–1253. 25201358 10.1016/S1470-2045(14)70377-8

[11] Glynne-Jones R Counsell N Quirke P Chronicle: results of a randomised phase III trial in locally advanced rectal cancer after neoadjuvant chemoradiation randomising postoperative adjuvant capecitabine plus oxaliplatin (XELOX) versus control. *Ann Oncol* 2014; 25:1356–1362. 24718885 10.1093/annonc/mdu147

[12] Rödel C Liersch T Becker H Preoperative chemoradiotherapy and postoperative chemotherapy with fluorouracil and oxaliplatin versus fluorouracil alone in locally advanced rectal cancer: initial results of the German CAO/ARO/AIO-04 randomised phase 3 trial. *Lancet Oncol* 2012; 13:679–687. 22627104 10.1016/S1470-2045(12)70187-0

[13] Beets GL Glimelius BL Adjuvant chemotherapy for rectal cancer still controversial. *Lancet Oncol* 2014; 15:130–131. 24440476 10.1016/S1470-2045(14)70016-6

[14] Bosset JF Calais G Mineur L Fluorouracil-based adjuvant chemotherapy after preoperative chemoradiotherapy in rectal cancer: long-term results of the EORTC 22921 randomised study. *Lancet Oncol* 2014; 15:184–190. 24440473 10.1016/S1470-2045(13)70599-0

[15] Sainato A Cernusco Luna Nunzia V Valentini V No benefit of adjuvant Fluorouracil Leucovorin chemotherapy after neoadjuvant chemoradiotherapy in locally advanced cancer of the rectum (LARC): Long term results of a randomized trial (I-CNR-RT). *Radiother Oncol* 2014; 113:223–229. 25454175 10.1016/j.radonc.2014.10.006

[16] Breugom AJ van Gijn W Muller EW Adjuvant chemotherapy for rectal cancer patients treated with preoperative (chemo)radiotherapy and total mesorectal excision: a Dutch Colorectal Cancer Group (DCCG) randomized phase III trial. *Ann Oncol* 2015; 26:696–701. 25480874 10.1093/annonc/mdu560

[17] Hong TS Ryan DP Adjuvant chemotherapy for locally advanced rectal cancer: is it a given? *J Clin Oncol* 2015; 33:1878–1880. 25940719 10.1200/JCO.2015.60.8554

[18] Geva R Itzkovich E Shamai S Is there a role for adjuvant chemotherapy in pathological complete response rectal cancer tumors following neoadjuvant chemoradiotherapy? *J Cancer Res Clin Oncol* 2014; 140:1489–1494. 24849731 10.1007/s00432-014-1712-5 PMC11823718

[19] Maas M Nelemans PJ Valentini V Adjuvant chemotherapy in rectal cancer: defining subgroups who may benefit after neoadjuvant chemoradiation and resection: a pooled analysis of 3,313 patients. *Int J Cancer* 2015; 137:212–220. 25418551 10.1002/ijc.29355 PMC4957736

[20] Haynes AB You YN Hu CY Postoperative chemotherapy use after neoadjuvant chemoradiotherapy for rectal cancer: Analysis of Surveillance, Epidemiology, and End Results-Medicare data, 1998–2007. *Cancer* 2014; 120:1162–1170. 24474245 10.1002/cncr.28545 PMC3981916

[21] Khrizman P Niland JC ter Veer A Postoperative adjuvant chemotherapy use in patients with stage II/III rectal cancer treated with neoadjuvant therapy: a national comprehensive cancer network analysis. *J Clin Oncol* 2013; 31:30–38. 23169502 10.1200/JCO.2011.40.3188 PMC5950500

